# Fostered and left behind alleles in peanut: interspecific QTL mapping reveals footprints of domestication and useful natural variation for breeding

**DOI:** 10.1186/1471-2229-12-26

**Published:** 2012-02-17

**Authors:** Daniel Fonceka, Hodo-Abalo Tossim, Ronan Rivallan, Hélène Vignes, Issa Faye, Ousmane Ndoye, Márcio C Moretzsohn, David J Bertioli, Jean-Christophe Glaszmann, Brigitte Courtois, Jean-François Rami

**Affiliations:** 1 Cirad, UMR AGAP, TA A108/3, Avenue Agropolis, Montpellier F-34398, France; 2 ISRA/Ceraas, Route de Khombole, BP 3320, Thiès Escale, Senegal; 3 Embrapa Recursos Genéticos e Biotecnologia, C.P. 02372, CEP 70.770-900 Brasilia, DF, Brazil; 4 Universidade de Brasília, Campus Universitário, CEP 70.910-900 Brasília, DF, Brazil

## Abstract

**Background:**

Polyploidy can result in genetic bottlenecks, especially for species of monophyletic origin. Cultivated peanut is an allotetraploid harbouring limited genetic diversity, likely resulting from the combined effects of its single origin and domestication. Peanut wild relatives represent an important source of novel alleles that could be used to broaden the genetic basis of the cultigen. Using an advanced backcross population developed with a synthetic amphidiploid as donor of wild alleles, under two water regimes, we conducted a detailed QTL study for several traits involved in peanut productivity and adaptation as well as domestication.

**Results:**

A total of 95 QTLs were mapped in the two water treatments. About half of the QTL positive effects were associated with alleles of the wild parent and several QTLs involved in yield components were specific to the water-limited treatment. QTLs detected for the same trait mapped to non-homeologous genomic regions, suggesting differential control in subgenomes as a consequence of polyploidization. The noteworthy clustering of QTLs for traits involved in seed and pod size and in plant and pod morphology suggests, as in many crops, that a small number of loci have contributed to peanut domestication.

**Conclusion:**

In our study, we have identified QTLs that differentiated cultivated peanut from its wild relatives as well as wild alleles that contributed positive variation to several traits involved in peanut productivity and adaptation. These findings offer novel opportunities for peanut improvement using wild relatives.

## Background

Polyploidy means that two or more complete sets of chromosomes of the same (autopolyploid) or different (allopolyploid) genomes are present in the same nucleus. It is a prominent and significant process in plant evolution [1,2]. Polyploidy has been considered important in conferring adaptive value to some cultivated species by increasing the allelic diversity, maintaining genome-wide heterozygosity and allowing the emergence of novel phenotypic variation [3-6]. The stages of polyploid formation usually include reproductive isolation from the progenitors, resulting in severe genetic bottlenecks. However, as most polyploid species have been formed recurrently from their wild progenitors [7], a moderate level of polymorphism has been kept in polyploid plants. Peanut (*Arachis hypogaea *L.) is an allotetraploid (2n = 4x = 40) native from South America with an AB genome. In contrast to the recurrent formation of several polyploid species, the allopolyploid structure of cultivated peanut is likely derived from a single hybridization between two wild diploid species followed by chromosome doubling [8]. Consequently, its monophyletic origin and domestication effects have greatly narrowed the genetic basis of the cultigen.

The peanut primary gene pool comprises elite breeding lines and landraces of the cultivated species *A. hypogaea *and *A. monticola*, a closely related wild tetraploid species of cultivated peanut [9]. Although a large amount of phenotypic variation is conspicuous in this gene pool, only a limited level of DNA polymorphism between genotypes has been observed [10-14]. With the increase of the number of molecular markers, efforts have been invested for developing genetic maps [15,16]. QTLs for physiological parameters and yield component traits linked with drought tolerance have recently been reported [17]. However, there has still been little progress on the integration of molecular markers in intraspecific peanut breeding programmes despite the challenge to obtain new varieties with resistance to diseases and tolerance to abiotic stresses.

The secondary gene pool of cultivated peanut mainly comprises wild diploid species (2n = 2x = 20) and represents an important source of novel alleles that can be used to improve the cultigen. Extensive work has been done to characterize genetic relationships between species of this gene pool and cultivated peanut using molecular markers [18-23] and cytogenetics [24-26]. Several wild diploid species have been hypothesized as the possible ancestors of the cultivated species. Recent studies have proposed *A. duranensis *(A genome) and *A. ipaensis *(B genome) as the most probable wild progenitors [24,27,28]. Favero *et al. *[29] produced a synthetic amphidiploid resulting from a cross between *A. ipaensis *and *A. duranensis *and doubling of the chromosome number. This amphidiploid has produced fertile hybrids when crossed with each of the botanical varieties of *A. hypogaea*. Furthermore, resistances to several diseases have been identified in wild species [30-33] and QTLs for disease resistance were recently mapped in a cross involving wild diploid species [34]. Introgression of disease resistance genes from the wild diploid species *A. cardenasii *into an elite peanut variety has also been reported [35,36]. However, the effective transfer of genes from peanut wild species to cultivated species was reported to be labor intensive [37] and the introgression of genes involved in the variation of complex traits such as yield has to our knowledge never been reported. Hence, genetic variation existing in wild species remains largely underexploited.

Wild relatives represent an important source of genes that has been successfully tapped to improve productivity and adaptation in various crops [38-40]. Tanksley *et al. *[41] have proposed an efficient advanced backcross-QTL (AB-QTL) approach to detect and map valuable QTLs and to simultaneously transfer them from wild to cultivated species. This approach has been widely adopted for mapping and introgressing QTLs involved in complex traits in several species [42].

QTL mapping in crosses between crops and their wild progenitors is also a powerful means for identifying genomic regions involved in morphological and physiological changes that distinguish crops from their wild relatives [43]. These morphological and physiological differences that have resulted from plant evolution under anthropogenic influences have been included in a generic term known as the "domestication syndrome" [44,45]. Features of the domestication syndrome have been shared in almost all agronomically important domesticated species. The targets of domestication include the loss of mechanisms for seed dispersal and dormancy, changes in plant growth habits and increases in the size of harvested plant parts [46-48]. Although pod dehiscence is absent in peanut, the long peg and isthmus observed solely in wild species have been identified as a potential mechanism for seed dispersal [49]. In cultivated peanut pods, the isthmus is virtually nonexistent and has given way to a more or less deep pod constriction that may represent a vestige of the isthmus. Cultivated peanut also displays a more compact growth habit compared to wild species. However a large range of variation still exists in cultivated species. Varieties belonging to subspecies *fastigiata *are characterized by an erect growth habit accompanied by traits such as loss of pod constriction and of seed dormancy. Prostrate growth habits are generally accompanied by small fruits with marked constriction and seeds demonstrating dormancy. These characters, which could be considered primitive, are mainly found in varieties belonging to the *hypogaea *subspecies [50].

Notwithstanding the identification of the two most probable wild progenitors of cultivated peanut, little is known about peanut evolution under domestication, and the genomic regions associated with domestication have never been reported. We recently published an SSR-based genetic map constructed using a BC_1_F_1 _population derived from a cross between the amphidiploid (*A. ipaensis *× *A. duranensis*)^x4 ^and a cultivated peanut variety, and an analysis of the genome-wide introgression of wild DNA fragments in the BC_2_F_1 _generation [51]. As a follow-up to this study, we have produced an AB-QTL population that represents a great opportunity to map QTLs involved in peanut domestication and to explore the reservoir of agronomically interesting alleles remaining in the wild species. As peanut is mainly grown under rainfed conditions in the arid and semi-arid tropics, it often faces moderate to severe drought conditions [52,53]. An important breeding objective is thus to develop varieties that can produce suitable yields under water-limited conditions.

In this article, we present a detailed QTL analysis of several traits involved in peanut productivity and adaptation under two water regimes as well as in the domestication syndrome. Based on these results, we report the identification of wild alleles that contribute positive variations to complex traits, we outline several regions of the peanut genome involved in the domestication process and we compare the distribution of QTLs in the subgenomes.

## Methods

### Population development

A population of 142 plants (87 BC_3_F_1 _and 55 BC_2_F_2_) was produced using 44 BC_2_F_1 _plants derived from the cross between the cultivated Fleur11 variety, used as recurrent parent, and the amphidiploid AiAd (*A. ipaensis *KG30076 × *A.duranensis *V14167)^x4 ^[29], used as donor parent. Fleur11, a local peanut variety grown in Senegal, is a Spanish type with an erect growth habit, low to moderate pod constriction, short cycle (90 days), high yielding, and tolerance to drought. The population was produced in greenhouse conditions at the Centre d'Etude Regional pour l'Amélioration de l'Adaptation à la Sécheresse (CERAAS), Thiès, Senegal. The breeding scheme for producing BC_2_F_1 _individuals has been described previously [51]. Each of the 44 BC_2_F_1 _individuals used as female parent was: i) backcrossed with Fleur11 and ii) allowed to self-pollinate. A total of 565 seeds were harvested and sown individually in large deep pots in the greenhouse. DNA was extracted from young seedlings and BC_3_F_1 _individuals were differentiated from BC_2_F_2 _individuals using 115 SSR markers that produced a total of 147 mapped loci. These loci were chosen to offer regular coverage of the genetic map produced previously [51]. All BC_3_F_1 _and BC_2_F_2 _individuals were allowed to self-pollinate to produce BC_3_F_2 _and BC_2_F_3 _families that were then used for the phenotyping experiment. The choice of the 142 individuals (i.e. 87 BC_3_F_1 _and 55 BC_2_F_2_) retained as the final population was based on two criteria: i) maximization of donor allele frequencies in heterozygous or homozygous situations at each of the 147 loci, and (ii) the number of seeds produced per BC_3_F_1 _and BC_2_F_2 _individual, which can be a strongly limiting factor in peanut.

### Field preparation

The experiment was conducted in the field from September to December 2009 at the Centre National de Recherche Agronomique (CNRA) in Bambey (14.42° N and 16.28W°), Senegal. In this research station, the soil is ferruginous, with 90% sand content, and low clay content (3-6%). Forty-five days before sowing, the field was plowed to eliminate weeds. One hundred and fifty kg/ha of organic fertilizer and 1 t/ha of mineral fertilizer (6-20-10) were added 4 weeks and 3 days before sowing, respectively. The field was kept manually weed-free before sowing and throughout the experiment.

### Experimental design

The 142 backcross families (BC_3_F_2 _and BC_2_F_3_) and the Fleur11 parent were tested under two water regimes: well-watered and water-limited. For each water regime, an alpha-lattice experimental design was used with two replications and nine blocks per replication. The blocks contained 16 rows, 3 m each. The individuals were arranged in rows of 10 plants. The spacing was 30 cm between plants and 50 cm between rows. Due to the limited number of seeds per BC family, one seed per hill was sown at 4 cm depth. Before sowing, the seeds were treated with Granox (captafol 10%, benomyl 10%, carbofuran 20%) to protect them from insects and diseases.

### Water management

In the geographical area of the Bambey research station, the rainy season lasts about 3 months, from early July to late September. The experiment was sown on 16^th ^September 2009. In both treatments (well-watered, water-limited), the total amount of water (rainfall + additional irrigation) received from the sowing date to 43 days after sowing (DAS) was 184 mm. After this date, corresponding to the pod filling stage, stress was applied in the water-limited treatment by withholding irrigation until 84 DAS, representing a total stress duration of 40 days. Irrigation was restarted from 84 DAS to harvest (95 DAS) and 21 mm of water was added. The total amount of water received in the water-limited treatment was 205 mm. In the well-watered treatment, irrigation was continued throughout the experiment until harvest, with 315 mm of water applied overall.

### Soil moisture status

The soil volumetric water content was measured every 4 days to a depth of 1 m at 10 cm intervals, from the sowing date to the end of the stress (84 DAS) in both treatments using a Diviner 2000 capacitance profile probe (Sentek Environmental Technologies, Stepney, Australia). In each treatment, the measurements of 12 access tubes were averaged. The access tubes were randomly scattered in the treatment plots. Variations in the soil water availability at each depth from 10 cm to 1 m was expressed as a fraction of transpirable soil water (FTSW) using the following formula:

$$FTSW\;(\%)\;=\;(SWC_{ERD}-SWC_{PWP})/(SWC_{FC}-SWC_{PWP})$$

where SWC_ERD_, SWC_PWP _and SWC_FC _correspond to the soil water content at effective rooting depth, permanent wilting point, and field capacity, respectively [54]. The field capacity value was obtained from a previous study carried out in Bambey [55].

### Trait evaluation

Except for the days to flowering, the plant growth habit and the main stem height, all traits were recorded after harvest. The plants were harvested row by row 95 days after sowing and exposed to ambient temperature (30-35°C) for 1 month to allow complete drying of haulms and pods. A total of 27 traits (Table 1) were evaluated in BC_3_F_2 _and BC_2_F_3 _families under one or two water regimes. The phenotypic value of each trait for each BC_3_F_1 _and BC_2_F_2 _individual was obtained by averaging the values of the corresponding BC_3_F_2 _and BC_2_F_3 _families. The trait values were then expressed on a per plant basis. As indicated above, due to the limitation in the seed number, one seed per hill was sown. The traits were thus evaluated on a minimum of 3 to a maximum of 10 plants (mean 8.5 plants) per family and per replication. Details on the trait measurements are given below.

**Table 1:** List of traits and descriptive statistics in well-watered and water limited treatments.

				Well-watered					Water limited				
					
Trait category	Trait name	Symbol	Unit	Mean	Fleur11	SD	h^2^	Stn	Mean	Fleur11	SD	h^2^	Stn
Flowering	*Days to flowering*	DFL	days	19.3	19.3	0.78	0.77	n	-	-	-	-	-
Plant architecture	*Growth habit*	GH	1-6 scale	5.2	6.0	0.62	0.81	n	-	-	-	-	-
	*Main stem height*	PH	cm	20.3	22.4	2.52	0.86	y	-	-	-	-	-
Pod morphology	*Pod width*	PWI	mm	10.9	11.3	0.66	0.74	n	12.6	12.7	0.8	0.87	n
	*Pod length*	PL	mm	26.8	27.7	1.73	0.71	y	28.5	28.0	1.7	0.86	y
	*Pod beak*	PB	0-9 scale	5.3	5.1	0.45	0.54	y	5.3	4.9	0.7	0.64	y
	*Pod constriction*	PC	0-9 scale	5.7	5.5	0.85	0.80	n	-	-	-	-	-
Seed morphology	*Seed length*	SL	mm	14.0	13.8	0.69	0.84	y	14.6	14.4	0.6	0.81	n
	*Seed width*	SWI	mm	8.6	8.8	0.45	0.82	n	9.2	9.2	0.4	0.81	n
Yield components	*Total biomass*	TB	g	94.6	98.6	10.37	0.54	n	78.1	75.7	6.8	0.50	y
	*Pod weight*	PW	g	19.6	19.6	2.66	0.39	y	17.1	15.3	3.4	0.57	y
	*Haulm weight*	HW	g	75.1	79.2	8.8	0.55	n	60.9	60.1	4.5	0.46	n
	*Seed weight*	SW	g	14.3	14.5	1.89	0.39	y	11.8	10.8	2.7	0.62	y
	*Shell weight*	SHW	g	5.3	5.2	0.97	0.48	y	5.9	5.2	0.8	0.50	y
	*Hundred pod weight*	HPW	g	108.5	117.0	12.5	0.80	n	98.8	108.9	13.9	0.79	n
	*Hundred seed weight*	HSW	g	54.6	60.2	5.34	0.77	n	53.3	54.7	6.3	0.90	n
	*Pod number*	PN	count	18.8	17.9	2.5	0.44	y	17.5	15.4	1.9	0.53	y
	*Seed number*	SN	count	34.3	32.5	6.57	0.48	n	32.2	29.9	4.3	0.51	n
	*Harvest index*	HI	%	22.8	22.3	2.18	0.39	y	22.5	21.6	1.7	0.44	n
	*Percentage of maturity*	PMAT	%	0.7	0.7	0.027	0.41	y	0.5	0.5	0.0	0.39	n

Fleur11: value measured for the cultivated parent, SD: standard deviation, h^2^: heritability, Stn: Shapiro-Wilk test for normality

#### Days to flowering

The number of days from sowing to flowering (**DFL**) was evaluated on the basis of the first flower appearance date.

#### Plant architecture

The plant growth habit (**GH**) and main stem height (**PH**) were recorded in the well-watered conditions at the podding stage and 60 days after sowing, respectively. The plant growth habit was recorded on a 1-6 scale using the descriptors for groundnut [56] where, 1 = procumbent 1, 2 = procumbent 2, 3 = decumbent 1, 4 = decumbent 2, 5 = decumbent 3, and 6 = erect. The main stem height was measured from the cotyledonary axil up to the terminal bud.

#### Pod morphology

Pod beak (**PB**), constriction (**PC**), length (**PL**) and width (**PWI**) were evaluated on 30 pods. All traits were measured in the two water regimes, except for pod constriction, which was measured in the well-watered condition only. Pod beak and constriction were recorded on a 0-9 scale according to the descriptors for groundnut. Pod length and width were measured using a caliper with a digital display.

#### Seed morphology

Seed length (**SL**) and width (**SWI**) were evaluated on 30 seeds in the two water regimes using a caliper with a digital display.

#### Yield components

The yield components were determined in the two water regimes based on the pod, haulm and seed dry mass. The total biomass was first weighed to determine the total biomass per plant (**TB**). The pods were removed and weighed to determine the total pod weight per plant (**PW**). The haulm weight per plant (**HW**) was calculated as the difference between the total biomass and the total pod weight. The total number of pods per plant (**PN**) was determined and 100 pods were randomly sampled and weighed (**HPW**). The 100 pods were shelled and mature pods with a dark internal pericarp color were counted (**PMAT**). All seeds contained in the 100 pods were weighed and mature seeds were separated from immature seeds and counted. The weight of 100 seeds (**HSW**) was calculated as the weight of mature seeds divided by the number of mature seeds multiplied by 100. The total seed weight per plant (**SW**) and the total number of mature seeds per plant (**SN**) were estimated based on the total number of pods (PN), the number of mature seeds in 100 pods (NMSH) and the weight of seeds in 100 pods (SWH):

SW=(SWH/100)*PNandSN=(NMSH/100)*PN.

The shell weight (**SHW**) was computed as the difference between the 100 pod weight and the 100 seed weight relative to the total number of pods per plant:

SHW=((HPW-HSW)/100)*PN

The harvest index (**HI**) was calculated as a percentage of the pod weight to the total biomass.

Stress tolerance indices (STI) were calculated for the pod number (**STI-PN**), seed number (**STI-SN**), 100 pod weight (**STI-HPW**), 100 seed weight (**STI-HSW**), pod weight (**STI-PW**), seed weight (**STI-SW**), haulm weight (**STI-HW**) and total biomass (**STI-TB**) using the following formula:

$$STI=\left(Yis*Yiw\right)/{\left(Ỹm\right)}^{2}$$

with Yis = phenotype value of individual (i) for a given trait in the stressed condition, Yiw = phenotype value individual (i) for a given trait in the well-watered condition and Ỹm = trait mean of all genotypes in the well-watered condition [57].

### Statistical analysis

Qualitative data such as the plant growth habit (GH), pod constriction (PC) and beak (PB) were first transformed to quantitative data using the ratio of the frequency of a phenotypic class by the total number of observations. All statistical analyses were performed using the R statistical programming language [58]. Basic statistical analyses (mean and standard deviation) were calculated for each trait. The data normality was checked with the Shapiro test for normality. An analysis of variance (ANOVA) was performed to estimate the genetic and replication effects on each trait under each water treatment. This was done following a standard linear model with genotype, replication, block and interaction effects, as

$${\text{Y}}_{\text{ijk}}=\text{μ}+{\text{G}}_{\text{i}}\text{ + }{\text{r}}_{\text{j}}\text{ + }{\text{b}}_{\text{jk}}\text{ + }{\text{e}}_{\text{ijk}}$$

with Y_ijk _= observed value for a given trait, μ = mean of the population, G_i _= genotype effect, r_j _= replication effect, b_jk _= block within replication effect and e_ijk _= residual error. In addition, a combined analysis of variance for the two water regimes was performed following a standard procedure of a fixed model with genotype, water regime, replication, and block and interaction effects, as

$${\text{Y}}_{\text{ijkl}}\text{ = }\text{μ}\;+\;{\text{G}}_{\text{i}}+\;{\text{W}}_{\text{j}}+\;{\text{r}}_{\text{jk}}+\;{\text{b}}_{\text{jkl}}+\;{\text{G}}_{\text{i}}\text{*}{\text{W}}_{\text{j}}+\;{\text{e}}_{\text{ijkl}}$$

with Y_ijkl _= observed value for a given trait, μ = mean of the population, G_i _= genotype effect, W_j _= water regime effect, r_jk _= replication within water regime effect, b_jkl _= block within replication and water regime effect, G_i_*W_j _= genotype × water regime interaction and e_ijkl _= residual error.

Estimates of broad-sense heritability were calculated as

$${{\text{h}}^{2}}_{\text{b}}=\;{{\text{σ}}^{\text{2}}}_{\text{G}}/{{\text{σ}}^{\text{2}}}_{\text{G}}+{{\text{σ}}^{\text{2}}}_{\text{E}}\text{ with }{{\text{σ}}^{\text{2}}}_{\text{G}}=\left(\text{M}{\text{S}}_{\text{G}}-\text{M}{\text{S}}_{\text{E}}\right)/\text{r and }{{\text{σ}}^{\text{2}}}_{\text{E}}=\text{M}{\text{S}}_{\text{E}}$$

where σ^2 ^_G _is the genotypic variation, σ^2 ^_E _the residual variation, MS_G _and MS_E _the genetic and residual mean squares and r the number of replications.

Data for each water regime were analysed using a linear mixed model fitted with the R/lme4 software package. In the model, we considered replications and blocks within replications as fixed effects and genotypes as random effects. Best linear unbiased predictors (BLUP) were extracted from this model for each genotype and trait and used for the QTL analyses.

### Molecular analysis and QTL identification

A genetic map that had been previously produced [51] was used for the QTL analysis. A framework map of 147 loci covering all LG with one locus every 12 cM was derived from this map. These markers were used to genotype the 142 individuals of the population. Interspecific advanced backcross populations (BC_2_F_2_, BC_3_F_1_) carry the risk of a low frequency of the wild donor allele at some loci. To overcome this situation, we checked the genotypic composition of the population at each marker and assessed the number of individuals in each genotypic class (i.e. homozygous for the recurrent parent, heterozygous and homozygous for the donor parent). For some loci, the number of individuals homozygous for the donor parent was below 5. Genotypes at these markers were replaced by missing data. For the QTL identification, standard interval mapping (SIM) was performed with the Haley-Knott regression method using the R/qtl package [59]. Because of the specific family structure of our advanced backcross population, individuals in generation BC_3_F_1 _and BC_2_F_2 _were considered separately to calculate the genotypic probabilities at each 1 cM interval using the MDM and GRAFGEN softwares [60], taking the generation and the genotype observed at the flanking markers into accounts. The genotypic probabilities of both generations were then combined and the resulting file was considered as a unique F_2 _population and further imported into R/qtl. QTL detection for each trait and treatment, was performed using the following model:

$$\text{y = }\text{μ}\;+\text{β}\text{x}+\text{α}{\text{z}}_{\text{1}}+\text{δ}{\text{z}}_{\text{2}}+\text{ε}$$

where, y is the observed phenotype, μ the mean of the population, α and δ the additive and dominance effects of the putative QTL, respectively, z_1 _and z_2 _are the probabilities for QTL genotypes conditional to the flanking marker genotypes, βx the BC_2_F_1 _family covariate effect (44 levels), and ε the residual error.

A two-dimensional two-QTL genome scan method was also used to test for the presence of two QTLs in the same linkage group. This was applied in some particular cases when the LOD curve of the single QTL genome scan method displayed two distinct LOD peaks for a given linkage group. A LOD threshold value to indicate a significant QTL effect was determined for each trait using 1000 permutations with a genome-wise significance level of α = 5% [61]. The confidence interval estimates of the QTL location were obtained using the 1.5-LOD support interval method [62]. The proportion of phenotypic variance (*R*^2^) explained by each QTL was obtained by fitting a model including the QTL and the BC_2_F_1 _family covariate. The proportion of phenotypic variance (*R*^2^) explained by all detected QTLs for a given trait was obtained by fitting a model including all detected QTLs and the BC_2_F_1 _family covariate. Chi-square tests were used to assess whether the QTL number between subgenomes and distribution between homeologous LG fitted a 1:1 ratio. The graphical representation of the QTLs was obtained using Spidermap software (Rami, unpublished).

## Results

### Soil moisture status and stress intensity

The soil moisture measurement results showed that the calculated FTSW values were maintained between 0.80 and 0.50 in the well-watered treatment (Additional file 1, Figure S1). This range of FTSW is generally considered sufficient to keep plants out of stress [63]. In the water-limited treatment the FTSW values decreased gradually from 0.8 at 43 DAS to 0.15 at 84 DAS. We have divided this period in three different stress intensity levels -low, moderate and severe- (indicated in Additional file 1, Figure S1) corresponding to peanut reproductive stages R5-R6, R6-R7 and R7-R8 respectively, as described by Boote [64]. Moderate stress occurred from the end of seed formation to the beginning of pod maturity (R6-R7) and severe stress occurred from the beginning of pod maturity to harvest maturity (R7-R8). These intensity levels were characterized by the wilting of plants in the afternoon for the moderate stress conditions, and at mid-morning for the severe stress conditions.

### Trait variability and heritability

The phenotypic values were normally distributed for most of traits in both conditions (Additional file 2, Figure S2). The population mean for each trait in each condition tended to be skewed towards the phenotypic value of the recurrent Fleur11 parent (Table 1). A small range of variation (4 days) was observed for DFL, with values ranging from 18 to 21 days after sowing. Conversely, a high level of variability was observed for the morphological traits. The plant growth habit (GH) showed a wide range of morphologies, ranging from completely prostrate to totally erect. A similar range of variation was observed for PB and PC, i.e. from inexistent to very prominent and from inexistent to very deep, respectively. A wide range of variation was also observed for the yield component traits (Table 1). In general, genotypes that out-performed the recurrent parent in terms of pod and seed number had smaller pods and seeds. However, we observed some genotypes that had a better performance than the cultivated parent in terms of number of pods, while keeping a similar 100-seed weight. Transgressive segregation was not observed for HSW, for which the best genotypes were similar to the recurrent parent.

The comparison between the two water treatments showed that water stress had a negative impact on the R6-R8 developmental stages, corresponding to grain filling and pod maturity. SW and PMAT were consequently the most affected traits, with a population average reduction of 21.0% and 18.9%, respectively. Water stress also negatively affected TB, PW and HW, with reductions of 17.4%, 12.8% and 17.2%, respectively, and to a lesser extent HPW, HSW, PN and SN, with reductions of 8.9%, 2.4%, 6.8% and 6.0%, respectively. The harvest index values were similar in the two conditions.

The analyses of variance showed significant differences (P ≤ 0.001) between genotypes for each trait in the two water treatments. The estimated broad sense heritability values were similar in the two water treatments except for PW, HPW and SW for which they were higher in the water-limited treatment (Table 1). For TB, PW, HW, PN, SN, SHW, HI and PMAT, the heritability estimates were moderate, ranging from 0.39 to 0.54 in the well-watered treatment and from 0.39 to 0.62 in the water-limited treatment. Higher heritability estimate values were obtained for HPW, HSW, DFL and plant, pod and seed morphological traits ranged from 0.54 to 0.90 in both conditions (Table 1). The combined analysis of variance showed a significant genotype × water treatment interaction (P ≤ 0.05) for a few traits, including PW, SN, SHW and PMAT (data not shown).

### Correlation between traits

The phenotypic correlations between traits in the well-watered and water-limited treatments are shown in Table 2. The same trend was observed in the two water treatments. The highest values (up to 0.80) were obtained between TB and HW, between PW, PN, SN and SHW, and between HSW and HPW. HI was negatively correlated with HW but positively correlated with PW, PN, SW, SN and PMAT. The phenotypic correlations between pod and seed morphology related traits, and HSW and HPW ranged from 0.23 to 0.95. The highest correlations were obtained between SWI, HSW and HPW (Table 2). The main stem height (PH) was positively correlated with almost all traits.

**Table 2:** Phenotypic correlations between traits in well-watered (bottom left half) and water limited (upper right half).

	TB	PW	HW	PN	SW	HSW	HPW	SN	SHW	PWI	PL	PB	PC	SWI	SL	HI	GH	PH	PMAT	DFL
TB	*0.56*	**0.74**	**0.92**	**0.66**	**0.69**	**0.24**	**0.33**	**0.65**	**0.79**	0.20	**0.28**	0.18	-	0.16	ns	ns	-	-	ns	-
PW	**0.70**	***0.48***	**0.42**	**0.87**	**0.99**	**0.24**	**0.44**	**0.87**	**0.90**	0.12	0.12	ns	-	0.16	ns	**0.61**	-	-	**0.24**	-
HW	**0.95**	**0.45**	*0.58*	**0.39**	**0.37**	0.19	0.19	**0.39**	**0.56**	0.20	**0.31**	0.19	-	0.12	ns	**-0.42**	-	-	-0.15	-
PN	**0.58**	**0.88**	**0.36**	*0.53*	**0.87**	-0.15	ns	**0.88**	**0.75**	-0.19	ns	ns	-	-0.17	-0.16	**0.53**	-	-	0.22	-
SW	**0.67**	**0.97**	**0.43**	**0.87**	*0.53*	0.19	**0.44**	**0.87**	**0.82**	ns	ns	ns	-	0.12	ns	**0.64**	-	-	**0.34**	-
HSW	**0.23**	0.21	0.20	-0.17	0.18	*0.83*	**0.72**	ns	**0.36**	**0.66**	**0.51**	ns	-	**0.77**	**0.61**	ns	-	-	-0.16	-
HPW	**0.35**	**0.40**	**0.26**	ns	**0.38**	**0.80**	*0.75*	0.20	**0.44**	**0.54**	**0.35**	ns	-	**0.58**	**0.37**	**0.27**	-	-	**0.24**	-
SN	**0.59**	**0.84**	**0.37**	**0.89**	**0.84**	-0.16	ns	***0.46***	**0.75**	ns	ns	ns	-	ns	-0.14	**0.52**	-	-	ns	-
SHW	**0.69**	**0.91**	**0.47**	**0.77**	**0.82**	**0.27**	**0.41**	**0.73**	***0.46***	**0.31**	**0.29**	0.13	-	**0.26**	0.18	**0.40**	-	-	ns	-
PWI	0.19	0.16	0.17	ns	ns	**0.58**	**0.55**	ns	**0.27**	*0.77*	**0.26**	ns	-	**0.72**	**0.29**	ns	-	-	**-0.32**	-
PL	0.22	0.15	0.21	ns	0.12	**0.43**	**0.41**	ns	**0.26**	**0.23**	*0.76*	ns	-	0.25	**0.67**	-0.13	-	-	-0.18	-
PB	ns	ns	ns	ns	ns	-0.13	-0.15	ns	ns	-0.20	0.20	*0.60*	-	ns	ns	ns	-	-	ns	-
PC	ns	ns	0.13	ns	ns	ns	ns	ns	ns	-0.12	**0.37**	**0.39**	-	-	-	-	-	-	-	-
SWI	0.15	0.15	0.13	-0.20	ns	**0.79**	**0.72**	-0.12	**0.26**	**0.69**	**0.31**	-0.14	ns	*0.81*	**0.46**	ns	-	-	-0.18	-
SL	0.16	ns	0.17	-0.18	ns	**0.58**	**0.47**	-0.16	**0.24**	**0.29**	**0.64**	0.23	**0.42**	**0.52**	*0.82*	ns	-	-	-0.12	-
HI	-0.22	**0.51**	**-0.49**	**0.49**	**0.52**	ns	0.15	**0.43**	**0.39**	ns	ns	-0.12	-0.17	ns	-0.12	*0.42*	-	-	**0.38**	-
GH	ns	**-0.23**	ns	**-0.31**	-0.20	ns	ns	**-0.28**	**-0.24**	ns	ns	ns	0.12	ns	ns	-0.23	-	-	-	-
PH	**0.46**	**0.35**	**0.42**	**0.23**	**0.33**	0.17	**0.29**	**0.25**	**0.34**	0.17	0.18	ns	0.14	0.14	ns	ns	**0.42**	-	-	-
PMAT	ns	0.15	ns	0.15	0.20	ns	ns	-0.14	ns	**-0.25**	-0.21	ns	ns	-0.17	-0.20	**0.27**	0.12	0.12	***0.28***	-
DFL	ns	ns	ns	ns	ns	ns	ns	ns	ns	ns	ns	ns	ns	ns	ns	ns	ns	-0.15	ns	-

Values in italic along the diagonal represent the correlation between well watered and water limited experiment when applicable. Correlations in normal font are significant at 5% and correlations in bold are significant at 1%.

### QTL identification

**Figure 1:**
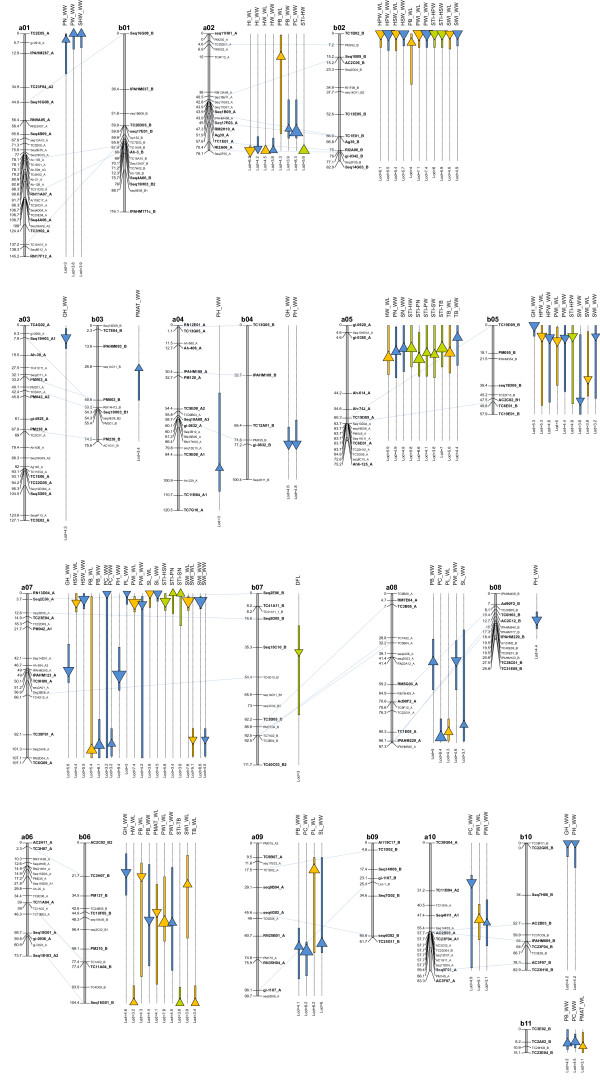
**Genetic map of detected QTLs**. Each QTL is represented by a triangle located at QTL peak and indicating the sign of the additive effect (upward: positive effect from the wild parent, downward: positive effect from the cultivated parent), and by a box representing the confidence interval. The size of triangle is proportional to the part of phenotypic variance explained by the QTL (*R^2^*). QTLs in orange were detected in water-limited treatment, QTLs in blue were detected in well-watered treatment and QTLs in green are stress tolerance indices QTLs. Locus names in bold represent the framework map used for QTL analysis.

A summary of QTLs detected in the two water regimes is provided in Table 3. At least one QTL was detected for each of the 27 traits analyzed, with a total of 95 QTLs mapped in the two environments (Figure 1).

**Table 3:** Characteristics of the QTLs detected for all traits in the two water treatments.

			Well-watered						Water-limited					
				
Trait Category	Trait Symbol	**L.G**.	Closest Marker	**Pos**.	**Conf. Int**.	Lod	**Add**.	R^2^	Closest Marker	**Pos**.	**Conf. Int**.	Lod	**Add**.	R^2^
Flowering	DFL	b07	Seq15C10_B	38	21-79	3.01	-0.74	9.3	.	.	.	.	.	.
Plant architecture	GH	a03	Seq19H03_A1	7.9	2-15	4.29	-1.19	14.1	.	.	.	.	.	.
		a07	TC9H08_A	50	33-58	5.56	-0.16	17.3	.	.	.	.	.	.
		b04	gi-0832_B	77.2	66.2-77.2	4.61	-0.71	14.1	.	.	.	.	.	.
		b05	TC19D09_B	0	0-7	5.27	-0.25	16.2	.	.	.	.	.	.
		b06	TC3H07_B	19	7-34	4.64	-0.46	13.9	.	.	.	.	.	.
		b10	TC22G05_B	0	0-9	4.17	-0.48	9.8	.	.	.	.	.	.
	PH	a04	TC9E08_A1	93	44-108	2.97	2.15	9.3	.	.	.	.	.	.
		a07	TC9H08_A	53	35-63	9.37	-0.94	26.7	.	.	.	.	.	.
		b04	gi-0832_B	77.2	61.6-77.2	4.82	-2.97	14.7	.	.	.	.	.	.
		b08	AC2C12_B	17.1	12-22.8	4.41	-2.56	13.8	.	.	.	.	.	.
		b10	TC22G05_B	0	0-16	4.2	-0.66	10.0	.	.	.	.	.	.
Pod morphology	PB	a02	RM2H10_A	62	43-68	3.93	0.22	8.5	seq11H01_A	15	1-65	4.33	0.28	12.6
		a07	TC38F01_A	99	82-107.1	6.14	0.16	17.4	TC6G09_A	102	2-107	5.38	0.42	15.4
		a08	RM5G08_A	44.7	29.7-62.7	5.05	0.34	13.1	.	.	.	.	.	.
		a09	RN25B01_A	67.5	55.5-88.5	4.14	0.35	11.6	.	.	.	.	.	.
		b06	TC19F05_B	50	14-80	4.35	-0.08	13.5	TC3H07_B	21.7	13-87	4.28	-0.56	12.5
		b11	TC2A02_B	9	0-13	4.16	0.45	12.7	.	.	.	.	.	.
		b02	.	.	.	.	.	.	TC1B02_B	5.9	0-32.9	4.04	0.30	11.8
	PC	a02	RM2H10_A	64	43-68	8.31	1.05	23.9	.	.	.	.	.	.
		a07	RN13D04_A	0	0-107.1	3.2	-0.69	10.0	.	.	.	.	.	.
		a07	TC38F01_A	98	88-106	3.2	0.72	9.8	.	.	.	.	.	.
		a08	TC1E05_A	93.7	81.7-96.1	6.39	0.61	19.0	.	.	.	.	.	.
		a09	RN25B01_A	70.5	64.5-86.5	8.19	1.10	23.6	.	.	.	.	.	.
		a10	AC2B03_A	25.6	21-81.3	4.9	-0.75	14.9	.	.	.	.	.	.
		b11	TC2A02_B	8.2	3-12	4.49	0.85	13.7	.	.	.	.	.	.
	PL	a07	RN13D04_A	0	0-7	2.97	-1.25	8.0	.	.	.	.	.	.
		a08	.	.	.	.	.	.	TC1E05_A	90.3	81.7-96.1	4.34	1.29	12.0
		a09	.	.	.	.	.	.	TC9B07_A	17.5	9.5-71.5	6.16	2.09	16.5
	PWI	a07	seq2E06_A	7	2-107.1	4.16	-0.57	12.2	seq2E06_A	6	2-12	7.39	-0.86	21.1
		a08	RM5G08_A	43.7	30.7-62.7	4.63	-0.77	14.4	.	.	.	.	.	.
		a10	TC11B04_A2	52	37-67	3.13	0.48	8.1	TC11B04_A2	50	40-63	5.12	0.41	15.1
		b02	TC1B02_B	0	0-8.9	7.35	-0.65	20.1	TC1B02_B	0	0-8.9	7.07	-0.78	20.3
		b05	TC19D09_B	8	0-43	4.39	-0.56	13.3	PM050_B	10	1-39	5.02	-0.89	14.9
		b06	TC19F05_B	52	16-83	4.49	0.35	12.8	TC19F05_B	52	22-64	7.86	0.55	22.3
Seed morphology	SL	a07	RN13D04_A	0	0-5	4.47	-0.13	12.5	RN13D04_A	0	0-9	3.85	-0.27	11.1
		a08	TC1E05_A	85.7	23.7-96.1	3.7	0.53	10.5	.	.	.	.	.	.
		a09	RN25B01_A	65.5	9.5-71.5	5.97	0.83	16.3	.	.	.	.	.	.
	SWI	a07	seq2E06_A	5	2-10	8.62	-0.51	23.7	seq2E06_A	5	2-11	7.56	-0.46	20.7
		a07	TC38F01_A	95	88-106	4.5	-0.46	8.7	TC38F01_A	95	88-106	5.1	-0.09	10.1
		b02	TC1B02_B	0	0-7.9	4.88	-0.35	14.2	TC1B02_B	0	0-9.9	4.59	-0.37	13.1
		b05	TC19D09_B	7	0-47.5	3.18	-0.32	9.5	seq19D06_B	35	4-46	3.81	-0.41	11.0
		b06	.	.	.	.	.	.	TC3H07_B	27	8-62	3.91	0.29	11.3
Yield components	HI	a02	RI2A06_A	75	67-76.1	4.18	-1.07	11.0	RI2A06_A	76.1	73-76.1	6.18	-1.77	18.1
	HPW	b02	TC1B02_B	0	0-10.9	5.5	-9.65	20.6	TC1B02_B	0	0-8.9	6.06	-12.38	17.0
		b05	TC19D09_B	8	0-50	4.63	-14.90	15.1	PM050_B	7	0-52	5.27	-18.94	15.0
	HSW	a07	seq2E06_A	3.7	0-10	5.53	-4.11	15.7	seq2E06_A	6	0-12	4.42	-4.86	12.4
		b02	TC1B02_B	0	0-7.9	5.72	-4.21	16.3	TC1B02_B	0	0-10.9	5.37	-5.64	14.9
	HW	a02	RI2A06_A	76	68-76.1	3.77	9.97	10.4	RI2A06_A	76.1	73-76.1	4.48	2.56	13.5
		a05	.	.	.	.	.	.	gi-0385_A	21	0-32	5.94	4.72	17.5
		b06	.	.	.	.	.	.	Seq18G01_B	104	93-104.4	3.18	3.76	9.7
	PMAT	b03	PM003_B	28.1	27-48.6	3.44	0.01	9.3	.	.	.	.	.	.
	PN	a01	TC2E05_A	4	0-26	3.03	1.96	9.3	TC19F05_B	44.9	27-92	4.11	-0.01	12.6
		a05	gi-0385_A	17	1-34	4.88	2.00	14.2	TC2A02_B	11	2-15	3.1	0.02	9.6
	PW	a01	TC2E05_A	0	1-10	3.82	2.60	11.7	.	.	.	.	.	.
	SHW	a01	TC2E05_A	0	0-9	3.87	0.92	12.6	.	.	.	.	.	.
	SN	a05	gi-0385_A	15	0-30	4.8	7.88	14.5	.	.	.	.	.	.
	SW	b05	TC19E01_B	49	6-57.9	3.58	-1.91	11.0	.	.	.	.	.	.
	TB	a05	gi-0385_A	8	0-26	4.42	11.79	13.2	gi-0385_A	18	0-31	5.63	7.00	16.6
		b06	.	.	.	.	.	.	Seq18G01_B	104.4	93-104.4	3.39	5.64	11.0
Stress tolerance indices	STI-HPW	b02	.	.	.	.	.	.	TC1B02_B	0	1-8.9	6	-0.16	16.8
		b05	.	.	.	.	.	.	TC19D09_B	7	0-51	4.86	-0.27	13.9
	STI-HSW	a07	.	.	.	.	.	.	seq2E06_A	5	0-11	5.64	-0.15	15.5
		b02	.	.	.	.	.	.	TC1B02_B	0	0-8.9	5.9	-0.16	16.2
	STI-HW	a02	.	.	.	.	.	.	RI2A06_A	76	73-76.1	5.57	0.07	16.4
		a05	.	.	.	.	.	.	gi-0385_A	15	0-27	5.85	0.17	17.1
	STI-PN	a05	.	.	.	.	.	.	gi-0385_A	22	4.6-33	6.6	0.19	19.4
		a07	.	.	.	.	.	.	RN13D04_A	0	0-13	3.38	0.16	10.4
	STI-PW	a05	.	.	.	.	.	.	gi-0385_A	18	0-33	4.14	0.16	12.3
	STI-SN	a07	.	.	.	.	.	.	RN13D04_A	0	0-21	3.57	0.22	11.0
	STI-SW	a05	.	.	.	.	.	.	gi-0385_A	19	0-34	3.78	0.13	11.5
	STI-TB	a05	.	.	.	.	.	.	gi-0385_A	15	0-27	7	0.17	20.1
		b06	.	.	.	.	.	.	Seq18G01_B	104.4	94-104.4	3.56	0.12	10.8

#### Days to flowering

One QTL for DFL detected on LG b07, explained 9.3% of the phenotypic variance (Figure 1). Interestingly, the flowering precocity was associated with the allele of the wild amphidiploid parent.

#### Plant architecture

A total of 11 QTLs, explaining individually 9.8% to 26.0% of the phenotypic variance, were involved in the variation of the plant architecture (Table 3). Five QTLs located on LGs a04, a07, b04, b08 and b10 were detected for PH. Fleur 11 alleles at QTLs on LGs b04, a07, b08 and b10 tended to increase the PH, but on LG a04 the PH increase was associated with the amphidiploid allele. The five QTLs together explained 40.9% of the phenotypic variance. Interestingly, two QTLs were located on homeologous regions on LGs a04 and b04 but the parental origin of the associated allele at these two QTLs differed (Figure 1). Six QTLs located on LGs a03, a07, b04, b05, b06, and b10 were detected for GH. At the QTLs for GH, all alleles conferring an erect growth habit phenotype were from Fleur11. Overall, they explained 46.1% of the phenotypic variance. We found a colocalization of QTLs for PH and GH in three regions on LGs b04, a07 and b10.

#### Pod morphology

A total of 31 QTLs were detected for traits related to pod morphology when considering the two water treatments. These QTLs individually explained 8.5% to 23.9% of the phenotypic variance (Table 3). Among the 10 QTLs detected for PB, six QTLs located on LG a02, b06 and a07 were detected in the two conditions, three on LGs a08, a09 and b11 were specific to the well-watered treatment and one on LG b02 was specific to the water-limited treatment (Figure 1). Amphidiploid alleles at QTLs on LGs a02, a07, a08, a09, b02 and b11 tended to confer a prominent beak, while on LG b06 the increase in beak prominence was associated with the allele of Fleur11. Overall, QTLs for PB explained 42.8% and 38.2% of the phenotypic variance in the well-watered and water-limited treatments, respectively. Seven QTLs located on LGs a02, a07, a08, a09, a10 and b11 were detected for PC. Amphidiploid alleles at QTLs on LGs a02, a08, a09 and b11 were associated with the constriction depth. On LG a10 the constricted pod phenotype was associated with the Fleur11 allele. The two QTLs on LG a07 were in repulsion. Overall, QTLs involved in constriction depth explained 50.2% of the phenotypic variance. Among the 11 QTLs detected for PWI, 10 QTLs located on LGs a07, a10, b02, b05 and b06 were detected in the two water treatments and one on LG a08 was specific to the well-watered treatment (Figure 1). All QTLs explained 49.2% and 58.5% of the variation of this trait in the well-watered and water-limited treatments, respectively. Fleur11 alleles at QTLs on LGs a07, a08, b02, b05 were associated with the increase in pod width while on LGs a10 and b06 the pod width increase was associated with the amphidiploid alleles. Three QTLs were detected for PL. The Fleur11 allele at the QTL on LG a07, detected in the well-watered treatment only, was associated with the increase in pod length. Amphidiploid alleles at QTLs on LG a08 and a09, detected in the water-limited treatment only, were responsible for the pod length increase.

#### Seed morphology

A total of 13 QTLs were detected for traits related to seed morphology when considering the two water treatments. These QTLs individually explained 8.7% to 23.0% of the phenotypic variance (Table 3). Among the nine QTLs detected for SWI, eight were detected in the two treatments, with four QTLs located on LG a07, two on LG b02 and two on LG b05. The QTL detected on LG b06 was specific to the water-limited treatment (Figure 1). Overall, these QTLs explained 38.6% and 55.5% of the phenotypic variation in the well-watered and water-limited treatments, respectively. Surprisingly, about half of the QTLs detected for SWI were distributed on LG a07, with two QTLs at a proximal position near the seq2E06_A locus and two at a distal position near the TC38F01_A locus. For QTLs on LGs a07, b02 and b05, the allele of Fleur11 conferred superior seed width in the two water treatments. The seed width increase at the QTL on LG b06 was associated with the amphidiploid allele. Four QTLs were detected for SL. Two QTLs located on LG a07 were detected in the two water treatments with the favourable alleles coming from Fleur11, while two QTLs located on LG a08 and a09 were specific to the well-watered treatment with the favourable alleles coming from the amphidiploid.

#### Yield components

A total of 26 QTLs explaining 9.2% to 20.6% of the phenotypic variance were detected for the 11 yield component traits in the two water treatments (Table 3). The number of QTLs per yield component trait ranged from one to five. Among the three QTLs significant for TB, two QTLs located on LG a05 were detected in both water treatments and one on LG b06 was specific to the water-limited treatment (Figure 1). At all these QTLs, the TB increase was associated with the amphidiploid alleles. One QTL for PW and one for SW were detected on LGs a01 and b05, respectively, in the well-watered condition. The wild allele at the QTL for PW conferred an increase in pod weight of 2.6 g per plant. For SW, the positive effect was associated with the allele of Fleur11. Four QTLs were detected for HW: two on LG a02 which were consistent across water treatments, and two on LGs a05 and b06, which were specific to the water-limited treatment. At all these QTLs, the HW increase was associated with the amphidiploid alleles. Interestingly, QTLs for HW on LGs a05 and b06 were located in the same genomic regions as those for TB, suggesting that the increase in TB could be explained by the increase in HW. QTLs for HI detected on LG a02 were detected in the two water treatments and were associated with the alleles of Fleur 11. Two QTLs located on LGs a01 and a05 for PN and one on LG a05 for SN were specific to the well-watered treatment. On LG a01, a QTL for PN colocalized with the one for PW, and on LG a05 a QTL for SN colocalized with the one for PN (Figure 1). Wild alleles at QTLs for PN and SN were responsible for an increase in pod and seed number per plant, respectively. Four QTLs were detected for HSW and four for HPW. QTLs for HSW on LGs a07 and b02 and those for HPW on LGs b02 and b05 were consistent across water treatments. QTLs for HSW and for HPW explained about 35.6% and 42.3% of phenotypic variance, respectively, in both conditions, and the positive effects were associated with the allele of Fleur11. One QTL was detected for SHW and colocalized with QTLs for PW and SN on LG a01. The amphidiploid allele at this QTL was associated with the increase in SHW. The three QTLs that conferred an increase in the percentage of pod maturity (PMAT) were detected on LG b03 in the well-watered treatment, and on LGs b06 and b11 in the water-limited treatment. The positive effects at QTLs on LGs b03 and b11 were associated with the amphidiploid alleles, while the positive effect at the QTL on LG b06 was associated with the Fleur11 allele.

#### QTLs related to stress tolerance indices

A total of 13 QTLs were significant for the stress tolerance indices (STI): two for total biomass (STI-TB) on LGs b06 and a05, one for pod weight (STI-PW) on LG a05, one for seed weight (STI-SW) on LG a05, two for haulm weight (STI-HW) on LGs a02 and a05, two for 100 pod weight (STI-HPW) on LGs b02 and b05, two for 100 seed weight (STI-HSW) on LGs a07 and b02, two for pod number (STI-PN) on LG a05 and a07 and one for seed number (STI-SN) on LG a07. These QTLs individually explained 10.4% to 20.1% of the phenotypic variance (Table 3). In most cases, the STI related QTLs colocalized with the trait for which they were calculated. Some exceptions included QTLs for STI-PW, STI-PN and STI-SN on LG a05 and LG a07, respectively, where no QTLs for PW, PN and SN were apparently detected (Figure 1). The positive effects for QTLs for STI-HPW and STI-HSW were associated with Fleur11 alleles. For the other STI related QTLs, the positive effects were associated with the amphidiploid alleles.

### Subgenomic distribution of QTLs

**Figure 2:**
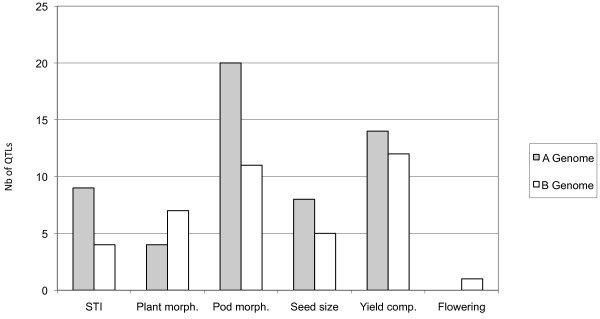
**QTLs distribution across subgenomes**. Each bar represent the number of QTLs detected in genome A (grey) and genome B (white) for each trait category (STI: stress tolerance indices, Plant morphology, Pod morphology, Seed size, Yield components, and Days to flowering).

When taking the two water regimes into account, the number of QTLs per trait and per LG varied from 1 to 11 and from 0 to 20, respectively. We did not detect QTLs on LG a06, b01 and b09. A total of 55 QTLs were mapped on LGs belonging to the A genome, with a maximum of 20 QTLs on LG a07. For LGs belonging to the B genome, a total of 40 QTLs were detected with a maximum of 11 QTLs on LGs b02 and b06. The number of QTLs detected per trait category and per genome is shown in Figure 2. The number of QTLs mapped on subgenomes (40 for the B genome versus 55 for the A genome) was not significantly different (P = 0.12). We found a subgenome specific QTL contribution for some traits, including PL and SL for which QTLs were detected only on the A genome (LGs a07, a08 and a09), and DFL, PMAT and HPW for which QTLs were detected only on the B genome (b02, b03, b05, b06, b07, b11). We found a significant difference in QTL distribution (P > 0.001) between homeologous LG. The most compelling examples were the difference in QTL number between homeologous LGs a06 and b06 (0 versus 11), and between homeologous LGs a07 and b07 (20 versus 1)(Figure 1). Furthermore, apart from QTLs for PB and PH that mapped to homeologous regions on LGs a02/b02 and a04/b04, all other QTLs for a given trait mapped to different homeologous LGs, thus indicating a marked inconsistency in QTL locations between homeologous LGs.

## Discussion

In our study, we developed an interspecific backcross population derived from a cross between Fleur11 and the amphidiploid between *A. ipaensis *and *A. duranensis*, which represent the most probable wild progenitors of allotetraploid cultivated species [24,27-29]. This material enabled us to map several QTLs for important agronomical traits under two water regimes, to explore the reservoir of useful alleles from the wild species, analyze the subgenome contribution to the quantitative trait variation and identify chromosomal regions associated with domestication.

### Peanut wild relatives represent a reservoir of useful alleles for peanut improvement

Peanut wild relatives have long been used as an important reservoir of disease resistance genes. Introgression of disease resistance genes into cultivated species using direct wild diploid × cultivated or via wild amphidiploid × cultivated crosses has been successfully used in peanut improvement [65-67]. However, the use of peanut wild relatives to dissect the molecular basis of more complex traits such as yield under both normal and water limited conditions has been impeded by the lack of molecular and mapping population resources.

In our study, we used an AB-QTL mapping approach to evaluate the genetic potential of wild species to enhance important agronomical traits in cultivated peanut. We detected 95 QTLs under the two water regimes. About half of the QTL positive effects were associated with amphidiploid alleles. These QTLs, which explained a large part of the phenotypic variance, contributed positively to valuable agronomic traits such as flowering precocity (9.0%), pod weight per plant (11.7%), pod number per plant (9.0% to 14.2%), seed number per plant (14.4%), pod size (8.0% to 22.0%), seed size (11.3%), pod maturity (9.5%) and biomass production (9.0% to 17.0%). Several QTLs involved in the increase in pod and seed size, pod maturity and biomass production were specific to the water-limited treatment.

We observed high consistency of QTLs across water treatments. For example, among the 25 QTLs for pod and seed sizes and yield components, 17 QTLs (68.0%) were consistent in terms of genomic location across water treatments. The stability of QTLs across the water treatments may be explained by the late occurrence of severe stress during the experiment, which led to low G × E and thus QTL × E interactions. However, the terminal stress in our experiment is representative of the most common drought events in the Sahel [54,68].

We used stress tolerance indices (STI) for yield components to decipher the molecular basis of yield performance under well-watered and water-stress conditions since these indices have been considered as good criteria for identifying genotypes that combine high yield and stress tolerance potential [57]. In addition, a positive correlation between STI for yield and yield under drought has been reported in peanut [53,69]. Thirteen QTLs were mapped for STI-related traits. Positive effects noted at nine QTLs were from the amphidiploid. Most QTLs mapped for STI related traits co-localized with the QTL of the trait for which they were calculated. This indicated a positive relationship between performances under the well-watered and water-limited treatments, as stressed above. Of special interest were STI-PN and STI-SN QTLs on LG a07, which mapped on a genomic region where no QTLs for PN and SN were mapped. Moreover, they co-localized with STI-HSW QTLs and several other QTLs involved in the increase in seed size under both well-watered and water-limited treatments. Favourable alleles at QTLs for STI-PN and STI-SN were from the amphidiploid while in this region positive alleles at all detected QTLs were from Fleur11. This suggests that this is a key region that could be involved in the trade-off between maintaining large sized seed and producing more seeds under water stress. In a cultivated allelic configuration, the production of large sized seeds could be favoured, while in a wild configuration it would be the seed number.

These results show that peanut wild relatives are valuable sources for improving important agronomical traits under both well-watered and water-limited conditions.

### QTLs for the same traits are mainly found in non-homeologous regions

In our study, the most striking results were the huge differences in QTL distribution on homeologous LGs and the lack of QTL consistency between homeologous LGs, while good colinearity has been reported between peanut A and B genomes [51,70,71]. QTLs for the same traits were mapped in 96% of cases in non-homeologous regions. Non-homeologous QTL locations could result from the lack of segregating alleles in one genome versus the other or by natural and/or human driven selection of different genes in the two subgenomes that contribute to the variation in the same trait. These results could also be explained by the differential control of gene expression in subgenomes and/or by movement of genes resulting in disruption of colinearity, as a consequence of interspecific hybridization and/or allotetraploidization.

Movement of genes resulting in disruption of colinearity has been reported in polyploid wheat [72] by homeologous BAC sequence comparison. Changes in gene expression, including genome specific gene silencing, unequal expression of homeologous genes, neofunctionalization or subfunctionalization of genes, have been extensively studied in various allopolyploids including wheat, cotton and brassica [73]. In cotton, high variation in the expression of the A and D subgenomes have been reported [74-76]. In addition, Rong *et al. *[77] reported that A and D subgenomes of the tetraploid cotton contributed QTLs for lint fiber development at largely non-homeologous locations. However, in hexaploid wheat, several authors have described the location of QTLs for the same trait on homeologous LG [78-80]. This suggests that variations in subgenome contributions to QTLs may depend on polyploid lineages.

### Clustering of key morphological trait QTLs: footprints of domestication

The marked phenotypic differences distinguishing crops from their wild progenitors are referred to as the domestication syndrome.

In our study, we considered that seed and pod size (SWI, SL, PWI and PL), 100 seed and pod weights (HSW, HPW), pod constriction (PC), and plant growth habit (GH) were traits that could be mostly involved in the peanut domestication syndrome. These traits were characterized by a high heritability ranging from 0.71 to 0.90. A total of 53 QTLs were detected for these traits. More than half of them clustered in three genomic regions on LGs a07 (11 QTLs), b02 (10 QTLs), b05 (8 QTLs). All QTLs with major effects for HSW and HPW, most of those for PWI, SWI, SL, and one for PC and GH were mapped in these three genomic regions. These QTLs individually explained 10.0% to 26.0% of the phenotypic variance and the favorable alleles were always from cultivated species. Other QTLs involved in PC and GH variations are found alone or in clusters with QTLs with lower effects for PWI, SWI, PL and SL on 10 different LGs. The high correlation between traits involved in pod size, seed size, HSW, HPW and the clustering of their associated QTLs suggests that one gene with pleiotropic effects or a limited number of linked genes are responsible for these trait variations in each region.

QTLs that greatly affected pod and seed size appeared to be clustered in three genomic regions while those affecting the plant and pod morphology seemed to be dispersed across the genome. Considering the number and distribution of QTLs for plant morphology and pod constriction on the genetic map and the primitive growth habits and constriction depths that still exist in peanut cultivated species, it is unlikely that these traits were the main focus of human selection in the incipient stages of domestication. Early stages of peanut domestication probably involved the fixation of alleles that increased pod and seed size at QTL clusters on LGs a07, b02 and b05. Preliminary modifications in plant growth habit and pod morphology could have started jointly with the increase in pod and seed sizes as QTLs for these traits were found in the same clusters. Subsequent improvements probably concerned the stacking of alleles involved in modification of the plant growth habit towards an erect type, in the modification of pod morphology, and the increase in pod and seed sizes at the other QTLs. These improvements, which could have taken place at different times and different locations, could be responsible for the morphological differences observed between peanut subspecies. Our findings on a limited number of domestication related QTLs and their clustering on specific areas of the peanut genome are in line with what has been reported for a wide range of crops, including tomato [81], maize [82], rice [83], wheat [84], and bean [46]. Our results could be further confirmed using a population with larger size, since at least in the case of sunflower many QTLs with small effects were mapped for domestication related traits [47].

Comparative QTL analysis is a powerful tool for unraveling the genetic basis of domestication in plant families. In cereals, particularly in maize, sorghum and rice, a small number of QTLs located in syntenic regions control some domestication traits [85]. In Solanaceae, Doganlar *et al. *[86] reported that 40% of the loci involved in eggplant fruit weight, shape and color have putative orthologous counterparts in tomato, potato and/or pepper. However, in legumes, Weeden [87] argued that although a similar number of genes have been modified during pea and common bean domestication, these genes were different. Nevertheless, these authors suggested that genes responsible for seed weight, photoperiod sensitivity and seed dormancy may involve homologous or orthologous sequences. Seed weight is one of the most important traits in legume domestication [48]. Comparative QTL analysis across legume species showed that QTLs for seed weight were located in orthologous regions of *Lotus *LG 2, soybean LG b1, pea LG I, and chickpea LG 8 [88]. Recent studies on legume synteny showed that *Lotus *LG2 shared common regions with *Arachis *LG 7 and LG 5 [89,90]. These two *Arachis *LGs were also collinear with LG a07 and b05 that carried the seed size QTL regions in our study. These results suggested that orthologous regions could be involved in genetic control of seed weight in peanut and several legume species. This could be further investigated by refining the syntenic relationships between these legumes species, and fine mapping and cloning gene(s) underlying the seed size QTLs.

## Conclusions

This manuscript reports a detailed QTL analysis of several traits involved in peanut productivity and domestication using a wild × cultivated advanced backcross population. We mapped a total of 95 QTLs in two water-treatments. Wild alleles contributed positive variation to many valuables agronomic traits such as flowering precocity, seeds and pods number, length and size as well as pods maturity. We also mapped peanut domestication related QTLs and proposed a temporal sequence of fixation of key traits during the domestication process that could explain morphological differences between peanut subspecies. In our study, we also showed that peanut A and B subgenomes contributed QTLs at largely non-homeologous regions as a probable consequence of the polyploidization. Our findings on the positive contribution of wild species to peanut improvement are in agreement with what has been reported for many other crops and offer novel opportunities for exploiting the reservoir of useful alleles remaining in peanut wild species.

## Authors' contributions

DF designed and coordinated the study, was involved in population development, field evaluation, genotyping data production, carried out data analyses and drafted the manuscript, HAT was involved in population development and field evaluation of the population, IF and ON were involved in field evaluation of the population, RR and HV were involved in genotyping data production, MCM contributed to the development of SSR markers used in this study, DJB and JCG were involved in the design of the study, BC contributed to editing of the manuscript and helped in data analyses. JFR conceived, designed and coordinated the study, was involved in data analyses, and editing of the manuscript. All authors have read and approved the final manuscript.

## Supplementary Material

Additional file 1 **Figure S1**. FTSW variation during population evaluation in well-watered and water-limited treatments. R5 to R8 correspond to peanut reproductive stages. I to III correspond to stress intensity levels. Click here for file

Additional file 2 **Figure S2**. Distribution of the traits measured in the population. The black arrow represents the value of the cultivated parent Fleur11. Click here for file
