# Supplementary figures and images for: Fostered and left behind alleles in peanut: interspecific QTL mapping reveals footprints of domestication and useful natural variation for breeding
Source: BMC Plant Biol. 2012 Feb 17;12:26. doi: 10.1186/1471-2229-12-26 (PMC3312858; doi:10.1186/1471-2229-12-26)

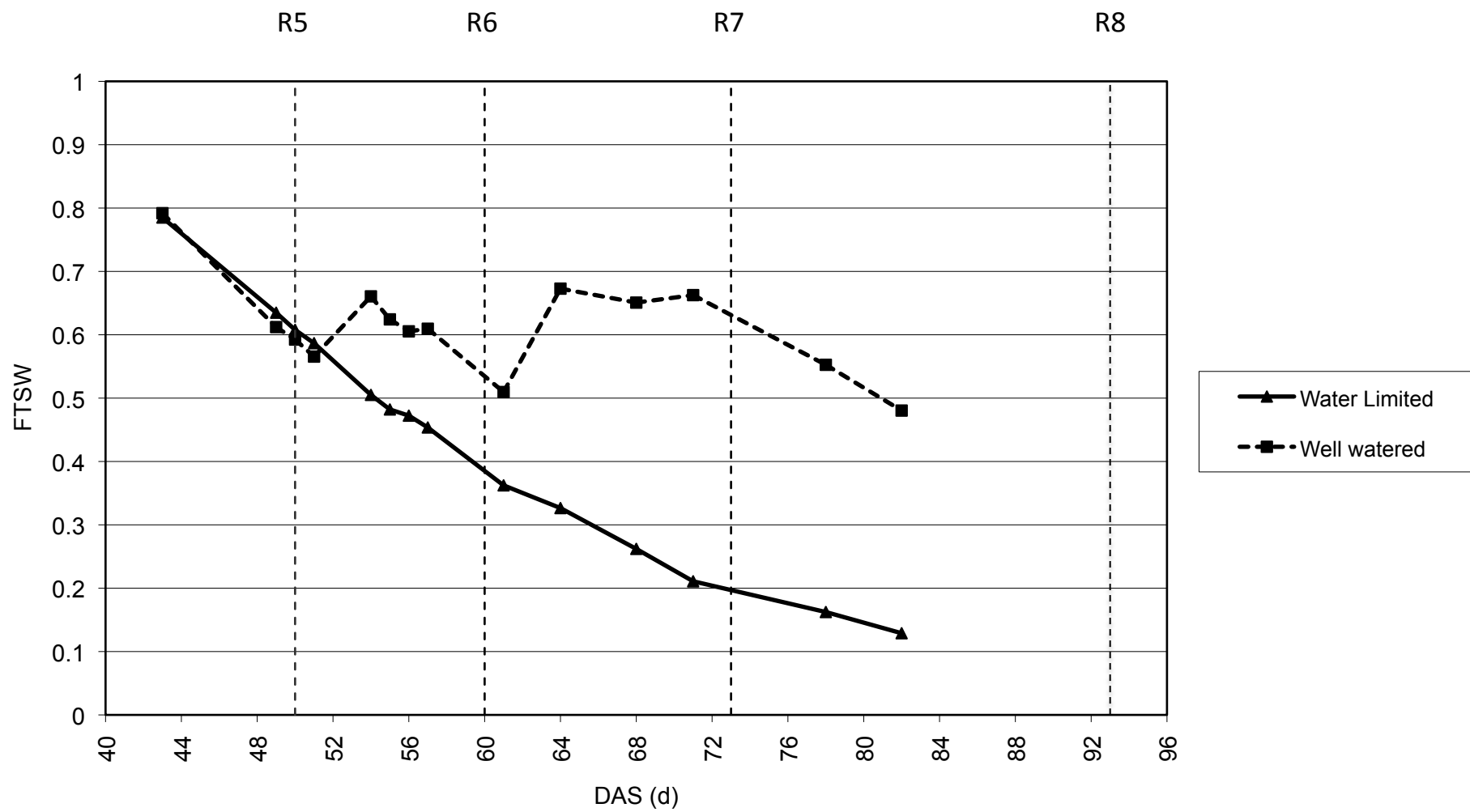

Supplement: Additional file 1 — Figure S1. FTSW variation during population evaluation in well-watered and water-limited treatments. R5 to R8 correspond to peanut reproductive stages. I to III correspond to stress intensity levels. [file 1471-2229-12-26-S1.PDF]

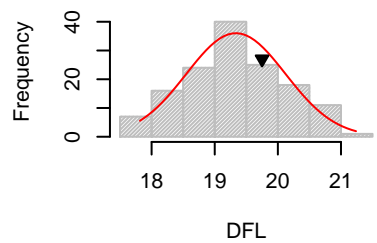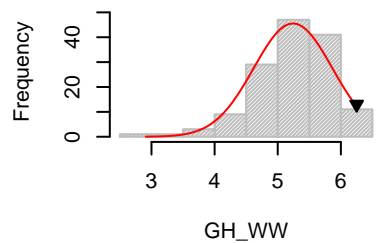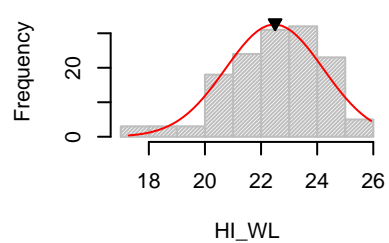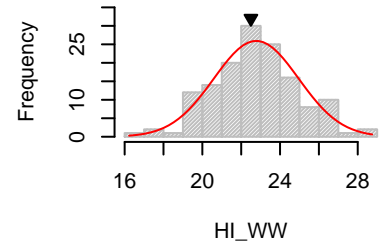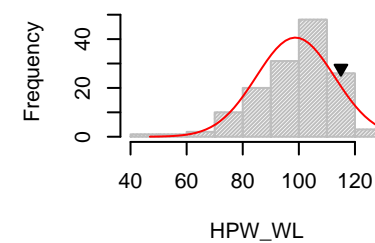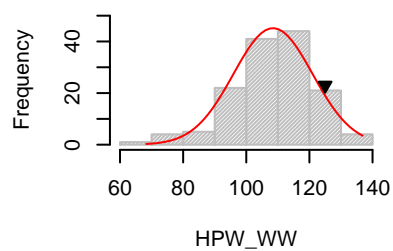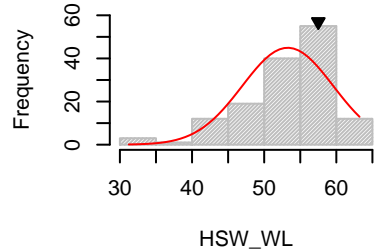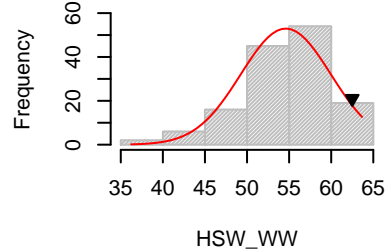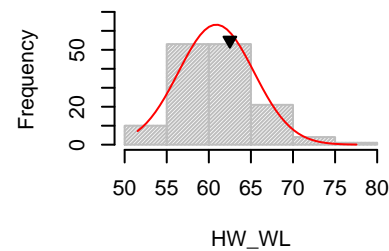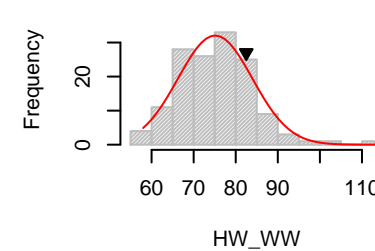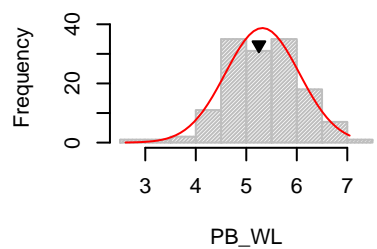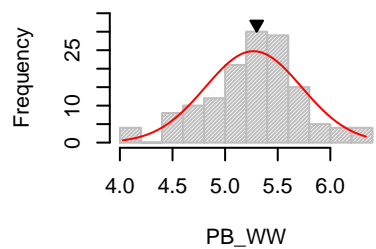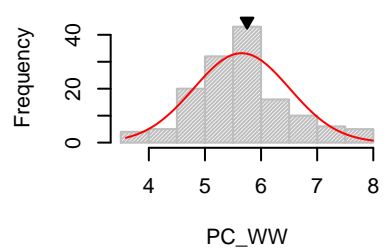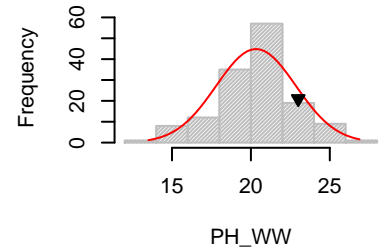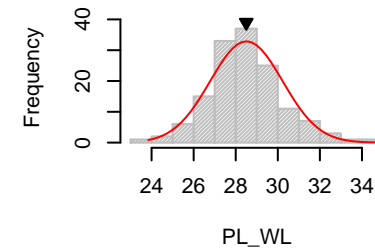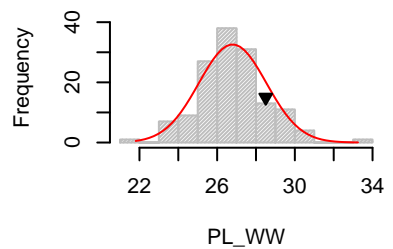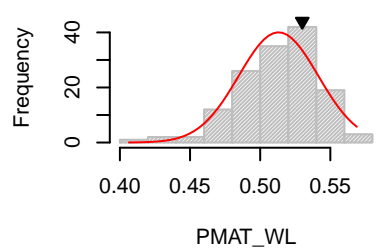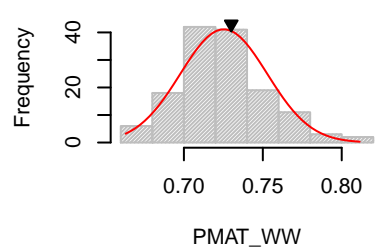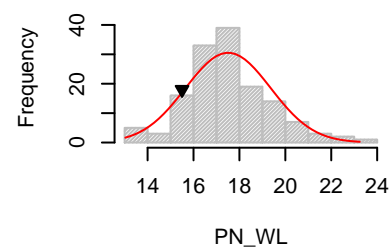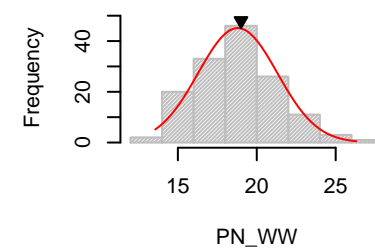

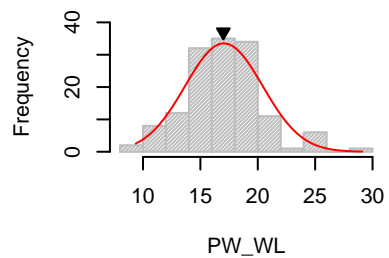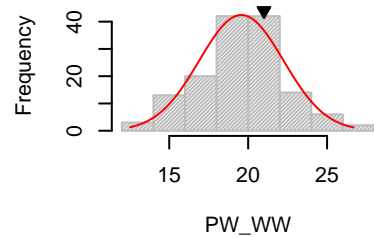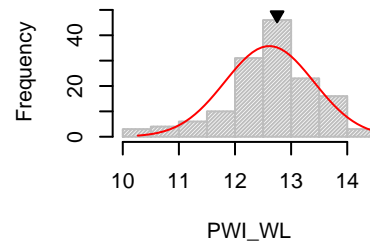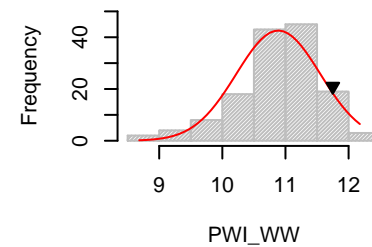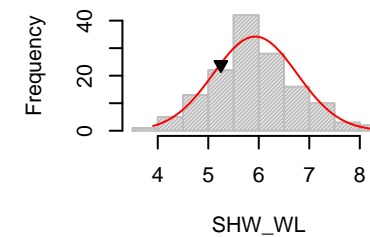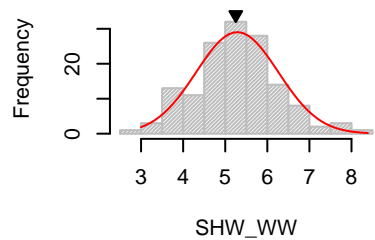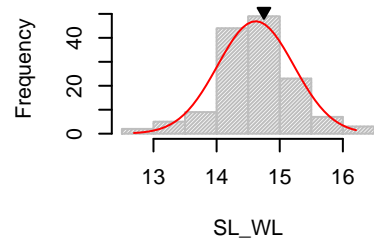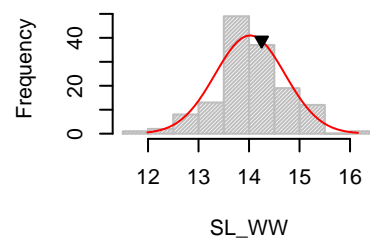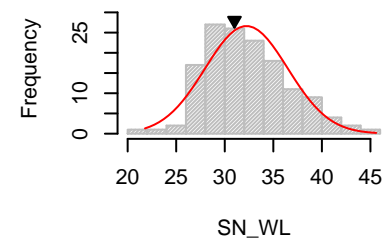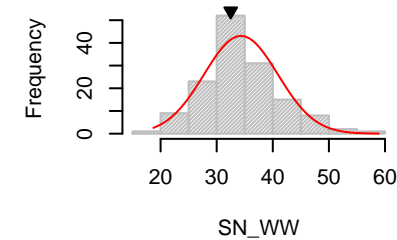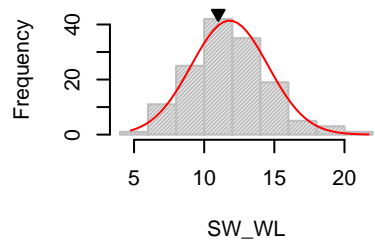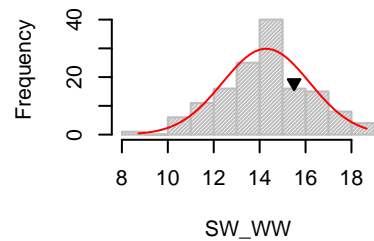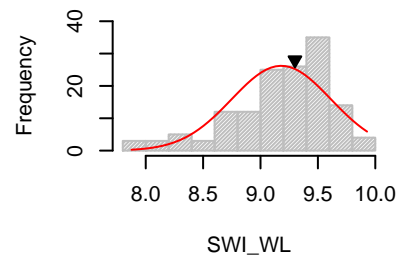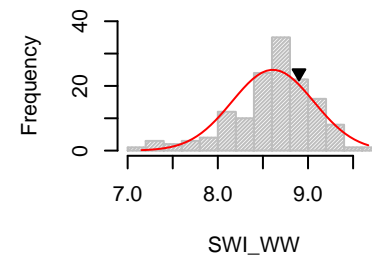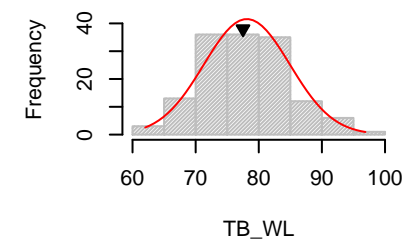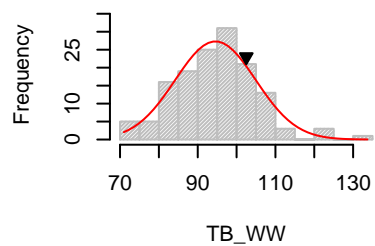

Supplement: Additional file 2 — Figure S2. Distribution of the traits measured in the population. The black arrow represents the value of the cultivated parent Fleur11. [file 1471-2229-12-26-S2.PDF]
